# Assessing gastroenterologist and patient acceptance of biosimilars in ulcerative colitis and Crohn's disease across Germany

**DOI:** 10.1371/journal.pone.0175826

**Published:** 2017-04-14

**Authors:** Emma Sullivan, James Piercy, John Waller, Christopher M. Black, Sumesh Kachroo

**Affiliations:** 1 Adelphi Real World, Manchester, United Kingdom; 2 Merck & Co., Inc., Kenilworth, NJ, United States of America; VU University Medical Center, NETHERLANDS

## Abstract

**Objectives:**

This study examined gastroenterologists’ motivation for prescribing biosimilars, assessed their treatment preferences in relation to prescribing behaviour, and explored patient attitudes to biosimilars.

**Methods:**

Data were taken from the Adelphi Real World Biosimilars Programme, a real-world, cross-sectional study undertaken in 2015–2016 with German gastroenterologists and patients with ulcerative colitis or Crohn’s disease. Gastroenterologists provided data on their prescribing behaviour and attitudes towards biosimilars, and invited the next eight eligible consecutive consulting patients to complete a detailed questionnaire. For analysis, gastroenterologists were split into ‘Investigative’, ‘Conservative’, and ‘Other’ groups.

**Results:**

Overall, 25 gastroenterologists and 136 patients participated. Biosimilars accounted for <15% of all biologic therapies and >80% of gastroenterologists would prescribe a bio-originator rather than biosimilar as 1^st^ line therapy if unrestricted. Patients showed some reluctance to accept biosimilars, although of those receiving biosimilars, 79% were satisfied with the current treatment of their condition, and 69% were satisfied with the control of symptoms. Although at least 35% of patients in each analysis group reported no concerns when starting treatment with a bio-originator or biosimilar, 41% of previously biologic-naïve patients prescribed a biosimilar indicated potential side effects and potential long-term problems, and 24% not knowing enough about the drug, as concerns.

**Conclusion:**

Results demonstrate that there is reluctance from patients to accept biosimilars and the need to further educate patients who are unsure to allow them to be involved in decision making, highlighting the importance of patient and physician communication. There remains a need for further research into non-clinical switching and the long term impact of prescribing biosimilars.

## Introduction

The World Health Organization defines biosimilars as ‘biotherapeutic products … similar in terms of quality, safety and efficacy to an already licensed reference biotherapeutic product’ [1]. For generic drugs, demonstration of identical structure and bioequivalence to the reference product is required for licensing purposes; however, biotherapeutic products (‘biologics’) are larger and more complex entities and this approach is not considered appropriate for biosimilars [1]. A biosimilar must therefore be developed strictly in accordance with procedures used for the reference product (or bio-originator), to ensure that no clinically meaningful differences exist between the ‘quality, safety and efficacy’ [2] or ‘safety, purity and potency’ [3], of the two.

In 2013, the European Medicines Agency’s (EMA) Committee for Medicinal Products for Human Use (CHMP) approved the marketing of two infliximab biosimilar compounds, for the same indications as the bio-originator [4]. Whilst Europe has historically been supportive of biosimilar products, this was the first example of a biosimilar monoclonal antibody being approved, and provided a defining moment for the use of biosimilars in a highly regulated market [4]. Both products were launched in major European markets, including Germany, in February 2015 [5]. Germany has seen some of the highest market shares in the European biosimilars market, with around 50% volume uptake reported [6]. Automatic substitution of bio-originators with biosimilars by pharmacists is not allowed in Germany [7] (this is mandated for generics [8]); however, recommended prescribing quotas have been set [9], although targets vary across regions [10].

There has been a paucity of published evidence regarding the impact of the launch of biosimilars on physician prescribing, but it would appear that, despite a high level of acceptance from a regulatory standpoint, there is still a marked reluctance from physicians to prescribe biosimilars [11,12]. It has been reported that, before prescribing a biosimilar, a physician requires reassurance that it has the same activity and safety profile as the bio-originator, and that the quality of the production process is guaranteed [13]–regulatory requirements in both Europe [2] and the US [3] ensure this, but concerns apparently remain. In one survey of European physicians across a range of disciplines, lack of a complete and accurate understanding of biosimilars, and the differences between generics and biosimilars, was identified [14], which might be one factor in the low acceptance levels by physicians.

As the active involvement of patients in treatment decisions is advocated [15], attitudes of patients to biosimilars is also important [16]; however, there is limited literature on this. One publication reported that in Europe and the USA showing only 6% of the general population have even a general impression of biosimilars, although significantly higher awareness (20%–30%) exists in patients diagnosed with a disease for which one or more biologic therapy is available and who are members of a patient advocacy group [17].

A biosimilar for etanercept was approved by CHMP in January 2016 [18], and approval of further biosimilars, a number of which will be treatment options in inflammatory bowel disease (IBD), is extremely likely over the next months and years. Biosimilars for adalimumab [19], certolizumab pegol [20], golimumab [21], ustekinumab [21], rituximab [22] are in development. To ensure optimum uptake of available products, it will be essential to have an in-depth understanding of physicians’ and patients’ perspectives with regard to biosimilars. In particular, it will be important to understand where non-clinical factors are playing a part.

The objectives of this study were to examine what motivates prescribing of biosimilars by gastroenterologists, to assess whether gastroenterologist preferences match actual prescribing behaviour, to explore IBD patient acceptance of biosimilars, and to understand patient satisfaction and concerns and compare how these relate to treatment with bio-originators and biosimilars.

## Methods

Data were drawn from the Adelphi Real World Biosimilars Programme (2015/16), a real-world, cross-sectional study based on completion of detailed record forms by physicians and a self-completion questionnaire by patients. Generation of the database was undertaken between December 2015 and March 2016 with gastroenterologists across Germany. The methodology was based on that used in the Adelphi Real World Disease Specific Programmes, which has been published previously [23].

### Study population and data collection

Gastroenterologists were identified by local fieldwork teams from panels of clinicians who had agreed to participate in survey research, and those meeting the eligibility criteria were invited to participate in the programme. Key eligibility criteria were: qualified as gastroenterologists for between 2 and 35 years; actively involved in management of patients with ulcerative colitis (UC) or Crohn’s disease (CD); and managing at least one patient with each disease in each of the following patient groups (see S1 Fig):

- BioSN: Patient receiving **bios**imilar who was previously biologic-**n**aïve.
- BioSE: Patient receiving **bios**imilar who has **e**xperience of a bio-originator.
- BioOA: Patient receiving **bio-o**riginator, initiated **a**fter February 2015.
- BioOB: Patient receiving **bio-o**riginator, initiated **b**efore January 2015.

Participating gastroenterologists provided data on their prescribing behaviour and attitudes towards biosimilars via a 30-minute online survey. They also recruited the next eight consecutive consulting patients aged 18 years or more, with a formal physician-confirmed diagnosis of UC or CD and being treated with either a bio-originator or biosimilar and who met the patient quota guidelines. The patient quota dictated that an equal number of patients was recruited from the patient groups. Each patient was invited to complete a patient self-complete form, containing detailed questions on demographics, current conditions, level of satisfaction and compliance with current treatment, patient-reported outcomes, and perspectives and opinions on using biologic therapies, including biosimilars and bio-originators.

### Analysis

Gastroenterologists were asked to indicate the relative importance to them of factors when making prescribing decisions. Based on their response, they were assigned to one of three groups for analysis:

1. Investigative: primarily concerned with symptom improvement and disease modification.
2. Conservative: primarily concerned with safety.
3. Other: influenced primarily by other factors.

Based on their prior experience of bio-originators and biosimilars, patients were split into the four groups indicated previously for analysis.

Specific objectives of the analysis were:

1. To highlight the prescribing behaviours of gastroenterologists who already prescribe biosimilars, to explore the factors that motivate them to do so, and to investigate whether these factors differ between gastroenterologists with different prescribing patterns.
2. To explore whether gastroenterologists encounter difficulties in convincing patients to accept biosimilars, and whether this differs between patients who were offered a biosimilar when biologic-naïve, those who were switched from a bio-originator to a biosimilar when a switch was indicated for clinical reasons and those who were switched from a bio-originator to a biosimilar for non-clinical reasons.
3. To assess patient understanding of, and satisfaction and concerns with, biologic treatment, and how these differ between those switched to a biosimilar from a bio-originator, those receiving a biosimilar who are biologic-naïve and those who remain on a bio-originator.

Descriptive statistics are presented: for numeric variables, the mean and standard deviation; for categorical variables, the number and percentage of subjects in each category.

### Ethics

Patients provided informed consent via a tick box on the front of the patient self-completion questionnaire. The data were collected according to market research guidelines; hence, no source validation was possible or required. Patient and doctor identities were not known to the analysis team. No identifiers were recorded for the patients; patient and physician forms for the same ‘matched’ patients were linked by unique numeric codes preprinted on the questionnaires.

## Results

A total of 25 gastroenterologists participated in this study, with 11 assigned to the ‘Investigative’ analysis group, 7 to the ‘Conservative’ group and 7 in the ‘Other’ group. Patient self-completion questionnaires were completed by 136 patients (69 CD, 67 UC), with 37, 33, 34 and 32 in the groups BioSN, BioSE, BioOA, BioOB, respectively.

### Prescribing behaviours

**Fig 1:**
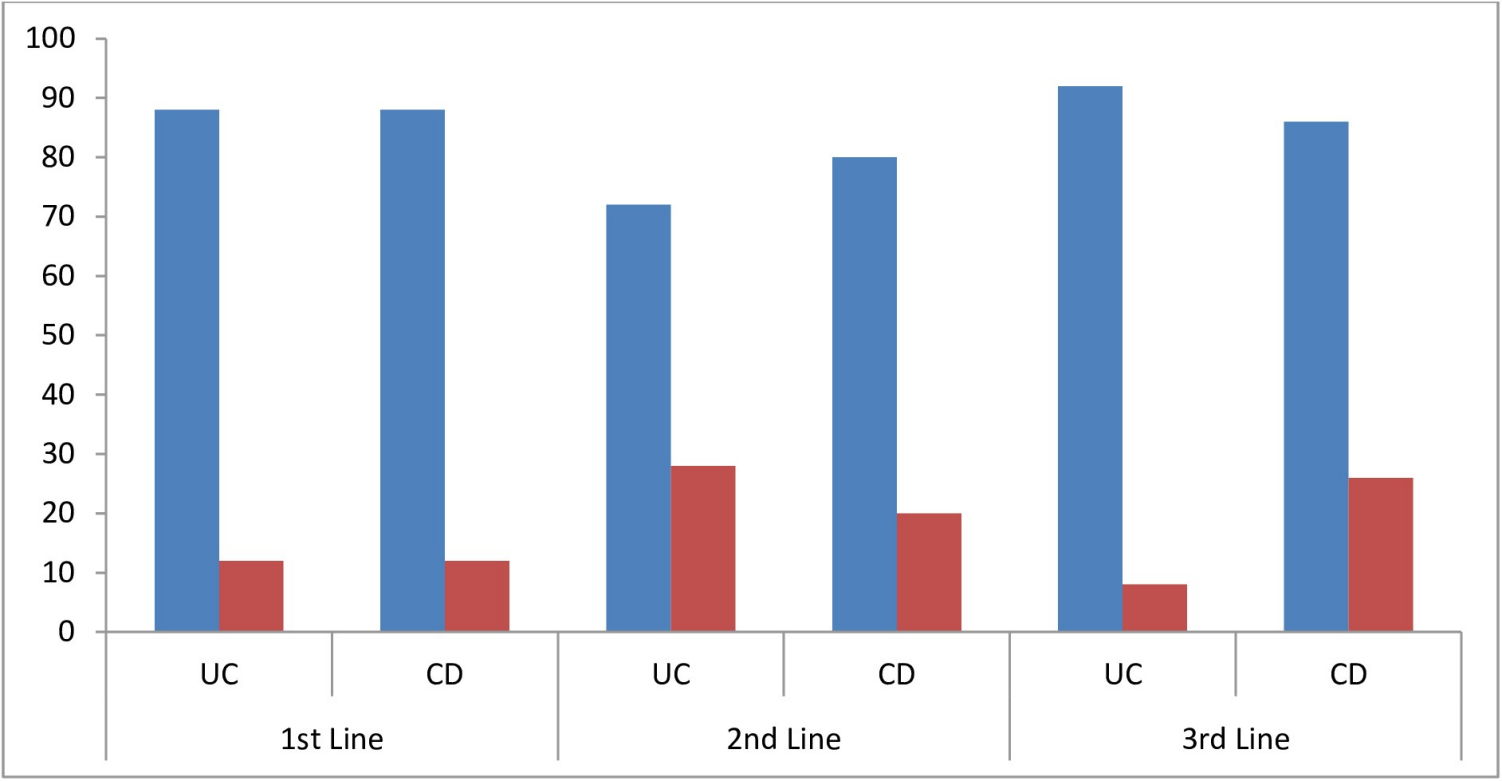
Gastroenterologist prescribing preference when it is assumed there are no restrictions or guidelines. “Blue”- Bio-originator, “red”—Biosimilar. CD, Crohn’s disease; UC, ulcerative colitis.

Gastroenterologists reported that biosimilars made up 12–13% of the biologic therapies and 4–5% of all drugs that they prescribed for the two conditions under study (**Table 1**). There was little expectation that this would increase over the following 12 months (**Table 1**). When asked about their preferences for prescribing under the assumption of unrestricted circumstances (i.e. no prescribing guidelines or other restrictions), 88% of gastroenterologists indicated they would prefer to prescribe a bio-originator to a biosimilar as 1^st^ line therapy for either UC or CD (Fig 1). This decreased to 72% and 80% for UC and CD, respectively for 2^nd^ line. When considering 3^rd^ line therapy, if no restrictions applied 92% of gastroenterologists would prefer to prescribe a bio-originator for UC, but only 76% for CD (Fig 1).

**Table 1:** Gastroenterologist-stated prescribing of biosimilars currently and expected in 12 months.

	Currently prescribing		Expect to be prescribing in 12 months	
	UC	CD	UC	CD
**N**	**25**	**25**	**25**	**25**
% of total prescribed drugs	4.2	5.0	4.1	5.1
% of total prescribed biologic therapies	12.1	13.2	12.4	14.7

CD, Crohn’s disease; UC, ulcerative colitis.

**Fig 2:**
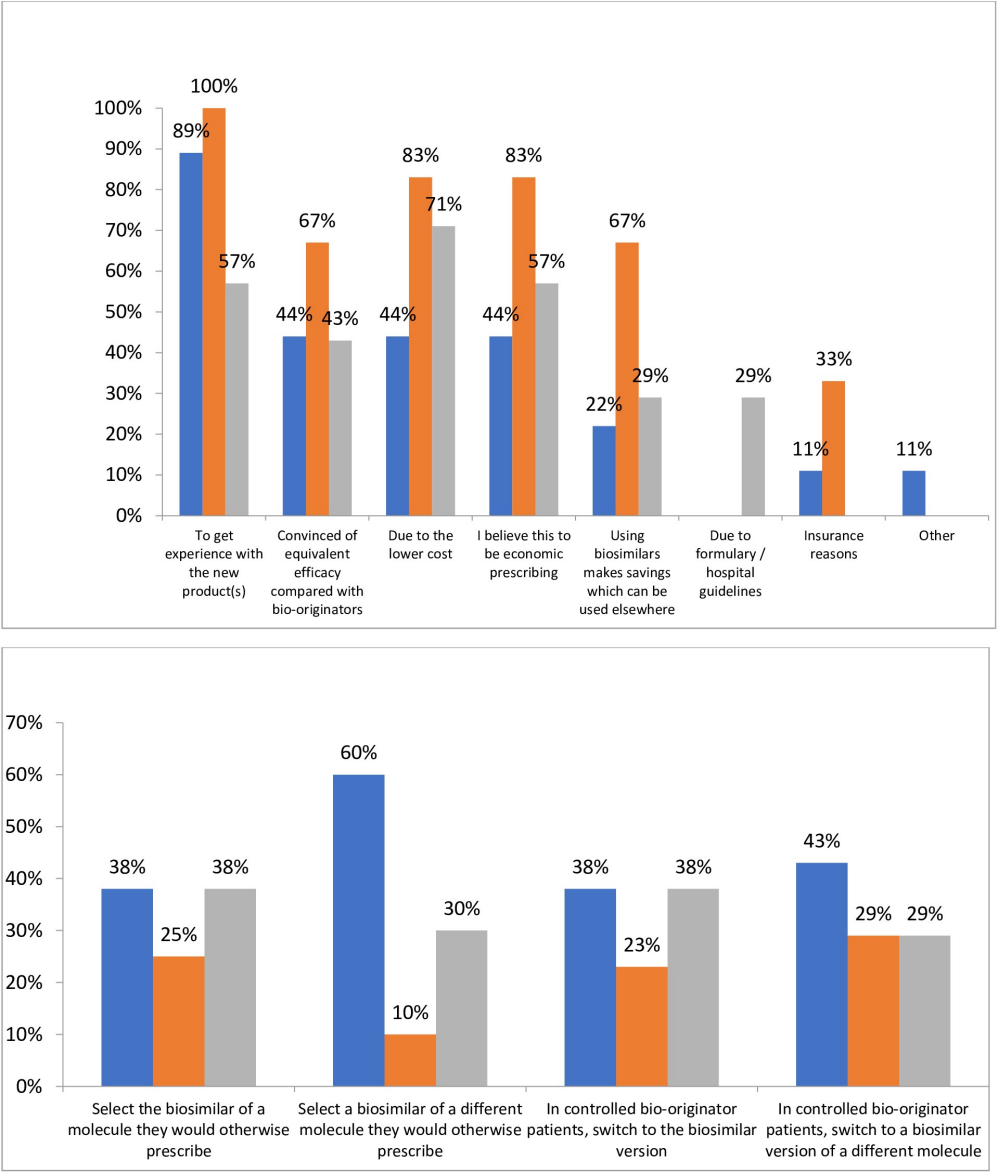
Gastroenterologists’ prescribing behaviours. (a) Reasons switched patients receiving bio-originators to biosimilars (b) Intended prescribing of biosimilars when more widely available. “Blue”—Investigative, “orange”—Conservative, “grey”–Other.

When asked their reasons for prescribing biosimilars instead of bio-originators, 89% of ‘Investigative’ and 100% of ‘Conservative’ gastroenterologists indicated that a desire to get experience with the new product(s) was a reason (Fig 2A). A belief that efficacy is equivalent to the bio-originator, lower cost and a belief that economic prescribing were all reasons selected by 44% of ‘Investigative’ gastroenterologists (Fig 2A). The lower cost and a belief that biosimilars reflect economic prescribing were both indicated as reasons to prescribe biosimilars instead of bio-originators by 83% of ‘Conservative’ gastroenterologists, while 67% of this analysis group indicated that both a belief that using biosimilars makes savings that can be used elsewhere and a belief that efficacy is equivalent to the bio-originator were reasons for the prescribing decision (Fig 2A).

In choosing multiple options from four responses to the question “*Once biosimilars are more widely available*, *how do you expect to use them?*”, the option chosen by most (60%) of ‘Investigative’ gastroenterologists was “*When a treatment change is required*, *I will select a biosimilar of a different molecule rather than the branded molecule I would otherwise have prescribed*”; this was selected by only 10% of ‘Conservative’ gastroenterologists (Fig 2B). “When a treatment change is required, I will select the biosimilar version of a molecule I would otherwise have prescribed” was selected by 38% of gastroenterologists in both the ‘Investigative’ and ‘Conservative’ analysis groups (Fig 2B).

### Patient attitudes

The mean percentage of patients reported by gastroenterologists to accept a biosimilar without reluctance, having never been prescribed a bio-originator or biosimilar, was 60%, compared with 43% of patients currently receiving a bio-originator and with no clinical reason for a change in therapy (Table 2). A small proportion of patients would not accept treatment with a biosimilar, but would accept the bio-originator; mean values across gastroenterologists were 10% for biologic-naïve patients, and 18% for those currently treated with a bio-originator and with no clinical indication for a therapy change (Table 2). Gastroenterologists reported that a small proportion (mean 8–9%) of patients refused any form of biologic therapy, regardless of the patient’s prior experience with biologic therapy or the reason for a switch in therapy (Table 2).

**Table 2:** Patient acceptance of biosimilars.

Patient status:		Biologic-naïve	Bio-originator patient in need of switch for clinical reasons	Bio-originator patient not in need of switch for clinical reasons
**% patients accepted without reluctance**	**N**	**24**	**24**	**24**
	Mean	60.6	50.4	43.3
	SD	27.8	27.9	27.5
**% patients reluctant but accepted as no other choice**	**N**	**24**	**24**	**24**
	Mean	21.0	25.6	29.4
	SD	17.1	18.4	18.3
**% patients refused but accepted bio-originator**	**N**	**24**	**24**	**24**
	Mean	10.2	15.8	18.0
	SD	11.3	17.9	14.4
**% patients refused biosimilar and bio-originator**	**N**	**24**	**24**	**24**
	Mean	8.1	8.1	9.3
	SD	9.2	12.9	10.6

N, number of gastroenterologists responding; SD, standard deviation.

### Patient satisfaction and concerns

**Fig 3:**
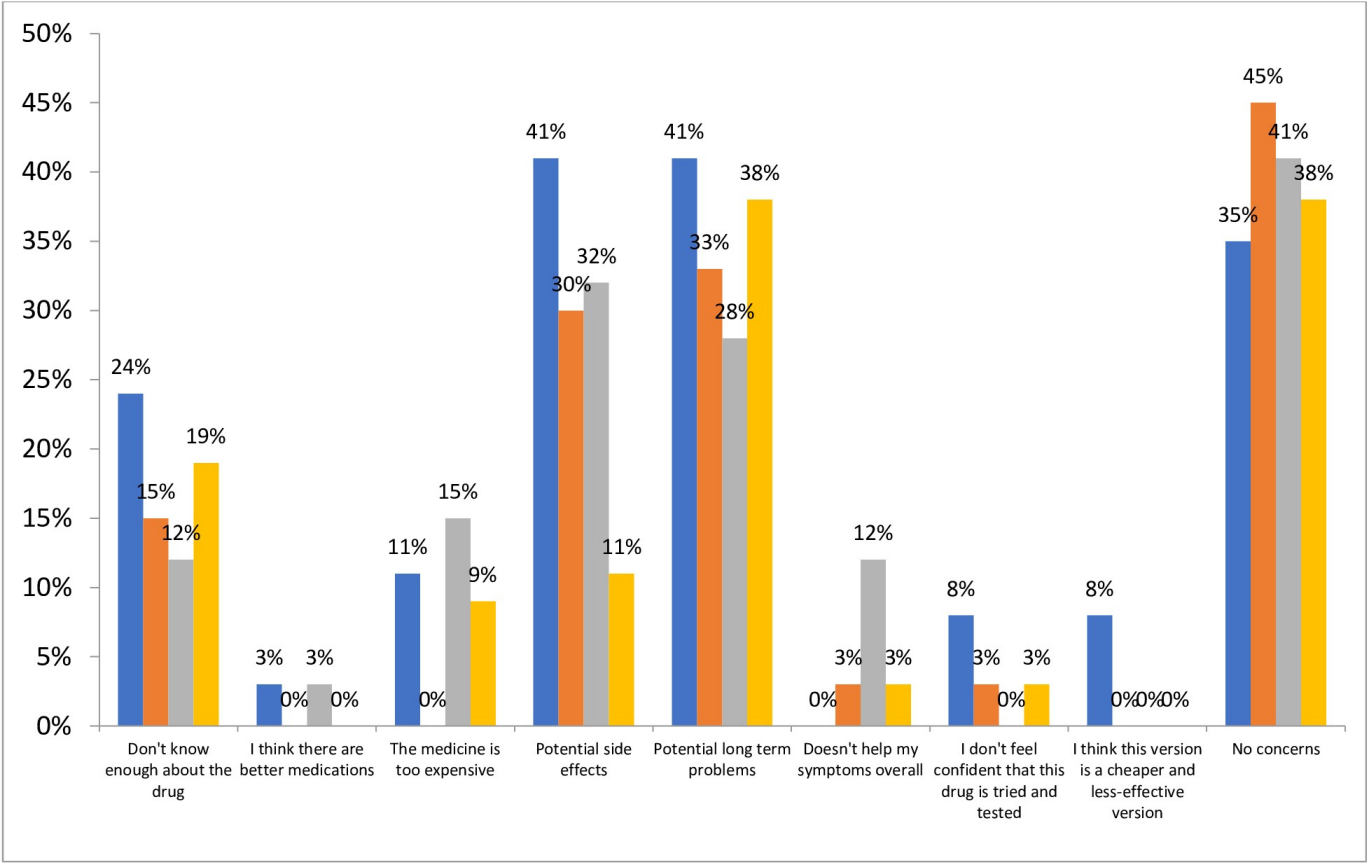
Patient satisfaction. (a) Satisfaction you are receiving the best treatment (b) Satisfaction with control of condition/symptoms by current treatment“Blue”—Biosimilar, “orange”- Bio-originator.

The majority of patients were satisfied with the current treatment of their condition, with 91% of patients receiving bio-originators satisfied, compared with 79% of those receiving biosimilars (Fig 3A). Satisfaction with the control of symptoms by current treatment was also high, with 87% and 69% of those on bio-originators and biosimilars, respectively, either satisfied or very satisfied (Fig 3B).

**Fig 4:**
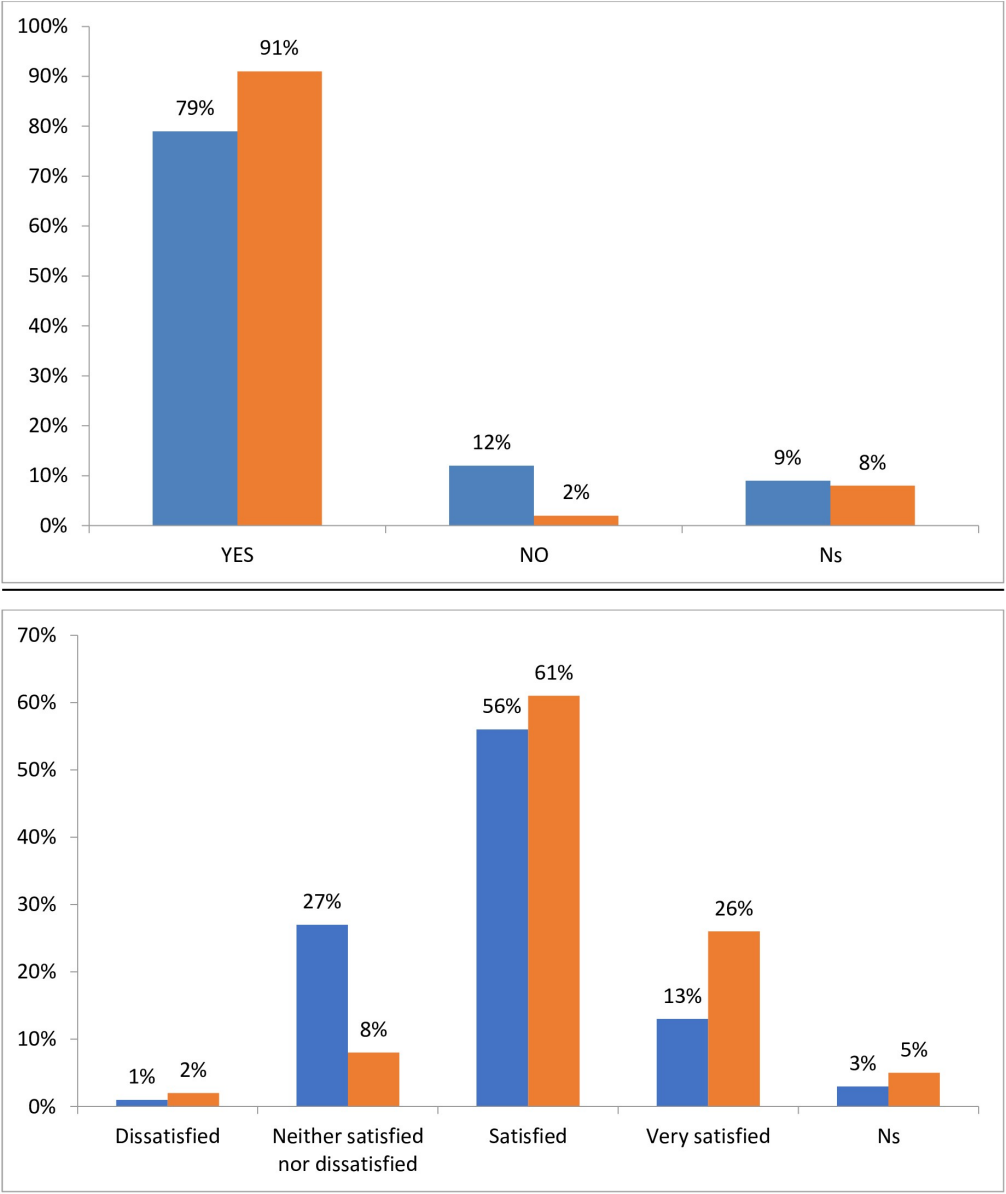
Patient concerns when first prescribed their treatment. BioSN, Patient receiving biosimilar who was previously biologic-naïve; BioSE, Patient receiving biosimilar who has experience of a bio-originator; BioOA, Patient receiving bio-originator who was initiated after February 2015; BioOB, Patient receiving bio-originator who was initiated before January 2015. “Blue”- BioSN, “orange accent 6”- BioSE, “grey”- BioOA, “orange”—BioOB.

When patients were asked what concerns they had when first prescribed their current treatment, at least 35% of those in each analysis group reported no concerns (Fig 4). However, 41% of patients prescribed a biosimilar who had not previously received any type of biologic indicated potential side effects and potential long-term problems as a concern, and 24% of this group were also concerned that they did not know enough about the drug (Fig 4). Of those patients prescribed a biosimilar who had previously received a bio-originator, 33%, 30%, and 15% were concerned about potential long-term problems, potential side effects and not knowing enough about the drug, respectively (Fig 4). For those receiving a bio-originator, a higher proportion of those whose treatment was initiated prior to January 2015 (i.e. before the approval of biosimilars) were concerned that they didn’t know enough about the drug, while a higher proportion of those whose treatment was initiated after February 2015 were concerned that the medication was too expensive (Fig 4).

## Discussion

We believe this to be one of the first reports of current and anticipated gastroenterologists’ prescribing habits and patients’ attitudes regarding biosimilars in IBD.

Reported levels of biosimilar prescribing were low, with little or no increase anticipated over the following 12 months. This may reflect the relatively recent introduction of biosimilars in the field of gastroenterology, as biosimilars for the treatment of chemotherapy-induced neutropenia, which have been available since 2008, have been reported to have 60% to 80% market share across the European ‘big 5’ countries [6]. A report published in March 2016 predicted that biosimilars will result in a highly competitive marketplace over the next five years [24].

For the majority of gastroenterologists classified as ‘Investigative’ based on factors influencing their prescribing decisions, a desire to gain experience using biosimilars was a reason for switching patients from a bio-originator to a biosimilar. This was also a reason for all gastroenterologists classified as ‘Conservative’ to switch therapy, but the majority of this group also considered other reasons in making a switch. As the methodology did not allow for probing to understand the reasons for particular responses, we cannot be certain whether gastroenterologists’ desire to gain experience using biosimilars reflects anticipation of increasing pressures from payers to prescribe biosimilars, but this is certainly a possibility. Cost-related reasons for switching patients to a biosimilar (including lower cost, a belief that biosimilars reflect economic prescribing, and a belief that using biosimilars makes savings that can be used elsewhere) were also indicated by large proportions of gastroenterologists in both the ‘Conservative’ and ‘Other’ groups. Participating gastroenterologists were required to be currently prescribing biosimilars at 1^st^ line; however a strong preference was indicated for using bio-originators as 1^st^ line therapy should no prescribing restrictions exist, suggesting a possible disconnect between gastroenterologists’ prescribing behaviours and preferences and some resistance to payer-driven guidelines.

The European Crohn’s Colitis Organization (ECCO) has undertaken two surveys of its members on their knowledge of, and attitudes towards, biosimilars. The first survey, revealed that gastroenterologists has little or no confidence in the use of biosimilars [25]. The main advantage of biosimilars was seen to be the lower cost, but respondents had concerns about the extrapolation of data across indication, as well as concerns regarding immunogenicity, safety and interchangeability. Following increased education on biosimilars, and more extensive use of biosimilar across many European countries in 2015, a further survey of to assess the evolution of ECCO members’ views on this topic was conducted in late 2015 [26]. Cost-sparing was still considered the main advantage of biosimilars by the majority (92%) of respondents, and immunogenicity was the main concern for 69% of respondents. A biosimilar was considered interchangeable with the bio-originator by 44% of respondents, an increase compared with 2013, when this figure was only 6%; however, 90% disagreed with automatic substitution by a pharmacist. Only 33% were against extrapolation across indications, as compared with 76% in 2013, and only 25% did not feel data should be extrapolated across different forms of IBD, compared with 53% in 2013. Confidence in the use of biosimilars had grown, with only 20% of respondents having little or no confidence in their use, compared with 63% in 2013.

In a survey of patients diagnosed with diseases with biologic treatment options together with members of the general public, 46–51% of respondents who were aware of biosimilars agreed that biosimilars were effective, with 43–47% agreeing that they were safe [17]. In the current study, some reluctance was observed in patients’ willingness to accept being prescribed a biosimilar, particularly among patients currently treated with a bio-originator and with no clinically-indicated reason for a switch of therapy. Few patients were concerned when first prescribed their treatment that there are better medications available, whilst concern about potential long-term problems was indicated by more than one-quarter of patients in each of the four analysis groups. Few patients in any of the analysis groups expressed cost-related concerns, although a slightly higher proportion of patients receiving a bio-originator initiated after the approval of biosimilars were concerned that the medication was too expensive, compared to those who were prescribed a bio-originator before biosimilars became available. As no further information is available for the reasons for any patient concerns, we cannot be certain if this is related to the information provided by the clinician at the time of prescribing.

The European Federation of Crohn’s and Ulcerative Colitis Association (EFCCA) investigated patients' perspectives concerning biosimilars [27]. A total of 1181 patients responded to a survey between November 2014 and October 2015; of these, 38% had heard of biosimilars. Only 25% of respondents had no concerns about biosimilars; 47% were concerned about safety, 40% about efficacy, and 35% that molecular basis of the biosimilar might be different from the bio-originator. Slightly more than half (56%) of respondents thought that lower cost should not come before safety and efficacy. Those patients responding wished to be informed and involved in decision-making concerning biosimilars; 66% would want to know whether they were receiving the bio-originator or the biosimilar, and 21% rejected the concept of interchangeability if the patient was not aware. These results indicate that patient and physician communication is important and further investigation may be required to determine the extent that additional factors are influencing the conversations. Potential factors such as volume of patients seen at hospital, care setting, clinician empathy may affect the time spent on discussing the prescribing of a biosimilar and such research will help determine how this impacts on patient outcomes. Only 31% of respondents expressed full confidence in biosimilars, even if they were prescribed and explained by the treating physician.

The concerns expressed in the various surveys reported above, as well as the current study, suggest that a cautious approach is needed until sufficient long-term data are available for biosimilars to mitigate any concerns that currently exist. The British Society for Rheumatology has made a number of recommendations related to biosimilars, including the need for biosimilars to undergo robust technology appraisals, safeguards against switching of patients responding well from a bio-originator to a biosimilar for non-clinical reasons, and the collection of detailed safety data for biosimilars [28]. In order to ensure traceability and accurate pharmacovigilance information for biosimilar products, more robust product identification mechanisms, including distinct non-proprietary names for biosimilars and bio-originators [29] and a requirement for the use of brand names on all prescriptions for biosimilars and bio-originators [28], have been recommended.

The current study explored the attitudes of gastroenterologists and their patients to biosimilars; this particular group has not had access to biosimilars until relatively recently. Rheumatologists have also only recently had biosimilars among their treatment options, with the approval of the two infliximab biosimilar compounds. A similar study to that described here has been conducted in the rheumatology field, and showed biosimilars accounting for <10% of all biologic therapies prescribed, with >95% of rheumatologists indicating they would prescribe a bio-originator rather than biosimilar as 1^st^ line therapy if no restrictions applied [30]. Patients expressed similar concerns to those of patients with UC and CD when starting treatment with a biologic therapy. By contrast, biosimilars have been used in oncology for some time, for treatment and prevention of chemotherapy-induced blood dyscrasias [31]. The approval of the infliximab biosimilars now gives oncologists a biosimilar option for treating cancer. A study exploring the attitudes of oncologists and their patients would be interesting; in particular to establish whether their attitudes differ from those in the fields of gastroenterology and rheumatology, given their experience of biosimilars as supportive therapy.

Some limitations of this study are acknowledged. The study was performed in Germany, which has an advanced healthcare system and broad access to treatment, which is not universally the case;[32],^,^[33],^,^[34] generalization of the findings beyond Germany warrants caution. Similar studies in other countries in western Europe might provide insight on differing attitudes, particularly when considering countries such as Denmark and Norway, where regulatory requirements have resulted in widespread use of biosimilar infliximab in place of the bio-originator [35]. Gastroenterologists were members of clinician panels maintained by fieldwork agencies, and as such all participating gastroenterologists were likely interested in survey research, and might not be fully representative of all practicing gastroenterologists. The eligibility criteria might have resulted in participating gastroenterologists being skewed towards those with a higher workload, and the requirement for gastroenterologists to be prescribing biosimilars as 1^st^ line treatment might have resulted in participants who are early adopters of new therapies with different perceptions of biosimilars to gastroenterologists who do not prescribe biosimilars. Only patients who presented to a gastroenterologist and agreed to participate were included; as a result the sample might include over-representation of patients who consult more frequently, and whose characteristics, perceptions, attitudes and concerns differ from the broader patient population. The number of patients included was rather low, and as such, caution should be used in extrapolating the findings to a wider patient population. Finally, the relatively low sample sizes precluded formal statistical analysis, and this was a cross-sectional rather than a longitudinal study; thus descriptive data are presented assessing the association between factors rather than an assessment of causality.

It is predicted that by 2020 biologics will form 28% of the global pharmaceutical market by value, with biosimilars offering potential savings of >50 billion euros across the European ‘Big 5’ countries and the USA [24]. The findings reported here support a number of publications [17,36,37] suggesting that education of physicians, patients, and other stakeholders, about biosimilars is essential to assist informed decision-making, and improve acceptance and use of biosimilars where appropriate. Further research with gastroenterologists and patients to further investigate their understanding, perspectives, and concerns of using biosimilars will help ensure that appropriate information is included in any educational initiative.

## Supporting information

S1 Fig Patient analysis groups based on current medication. This is the S1 Fig Legend.
(TIFF)

S1 Raw Data This is the raw data file for all analyses. (DOCX)
