# Supplementary material for: Assessing gastroenterologist and patient acceptance of biosimilars in ulcerative colitis and Crohn's disease across Germany
Source: PLoS One. 2017 Apr 14;12(4):e0175826. doi: 10.1371/journal.pone.0175826 (PMC5391967; doi:10.1371/journal.pone.0175826)
Supplement: S1 Raw Data — (DOCX) [file pone.0175826.s002.docx]

Raw Data Pertinent to ‘Assessing gastroenterologist and patient acceptance of biosimilars in ulcerative colitis and Crohn’s Disease across Germany’

| **S1 Table. Gastroenterologist-stated prescribing of biosimilars currently and expected in 12 months** | | | | |
| --- | --- | --- | --- | --- |
|  | Currently prescribing | | Expect to be prescribing in 12 months | |
|  | UC | CD | UC | CD |
| **n** | **25** | **25** | **25** | **25** |
| % of total prescribed drugs | 4.2 | 5.0 | 4.1 | 5.1 |
| % of total prescribed biologic therapies | 12.1 | 13.2 | 12.4 | 14.7 |

CD, Crohn’s disease; UC, ulcerative colitis

Question: What % of your patients with each condition are currently receiving each product? What % of your patients do you expect will be receiving each product in 12 months’ time?

| **S2 Table: Gastroenterologist prescribing preference when it is assumed there are no restrictions or guidelines** | |
| --- | --- |
| First. Line UC: | |
| Base | 25 |
| Bs, %(n) | 12 (3) |
| Bo, n | 88 (22) |
| Second. Line UC: | |
| Base | 25 |
| Bs, n | 28 (7) |
| Bo, n | 72 (18) |
| Third. Line UC: | |
| Base | 25 |
| Bs, n | 8 (2) |
| Bo, n | 92 (23) |
| First. Line CD: | |
| Base | 25 |
| Bs, n | 12 (3) |
| Bo, n | 88 (22) |
| Second. Line CD: | |
| Base | 25 |
| Bs, n | 20 (5) |
| Bo, n | 80 (20) |
| Third Line. CD: | |
| Base | 25 |
| Bs, n | 24 (6) |
| Bo, n | 76 (19) |
| CD, Crohn’s disease; UC, ulcerative colitis  Question: Assuming there were no restrictions on your prescribing or guidelines you needed to follow, what advanced therapy would you prefer to use first, second and third for each condition? | |

| **S3 Table. Gastroenterologists’ prescribing behaviours (a) Reasons switched patients receiving bio-originators to biosimilars** | | | | |
| --- | --- | --- | --- | --- |
|  | Base | Investigative | Conservative | Other |
| Base | 22 | 9 | 6 | 7 |
| I wanted to get experience with the new product(s), % (n) | 82 (18) | 89 (8) | 100 (6) | 57 (4) |
| I am convinced of equivalent efficacy compared with biologic originators, % (n) | 50 (11) | 44 (4) | 67 (4) | 43 (3) |
| Due to the lower cost, % (n) | 64 (14) | 44 (4) | 83 (5) | 71 (5) |
| I believe this to be economic prescribing, % (n) | 59 (13) | 44 (4) | 83 (5) | 57 (4) |
| Using biosimilars allows me to make savings which can be used elsewhere, % (n) | 36 (8) | 22 (2) | 67 (4) | 29 (2) |
| Due to formulary / hospital guidelines, % (n) | 9 (2) | - | - | 29 (2) |
| Insurance reasons, % (n) | 14 (3) | 11 (1) | 33 (2) | - |
| Other, % (n) | 5 (1) | 11 (1) | - | - |
| Question: Why did you prescribe the biosimilar(s) rather than the originator biologic(s)? | | | | |

| **S4 Table. Gastroenterologists’ prescribing behaviours (b) Intended prescribing of biosimilars when more widely available** | | | |
| --- | --- | --- | --- |
|  | Investigative | Conservative | Other |
| Select the biosimilar of a molecule they would otherwise prescribe, % | 38 | 25 | 38 |
| Select a biosimilar of a different molecule they would otherwise prescribe, % | 60 | 10 | 30 |
| In controlled bio-originator patients, switch to the biosimilar version, % | 38 | 23 | 38 |
| In controlled bio-originator patients, switch to a biosimilar version of a different molecule, % | 43 | 29 | 29 |
| Question: Once biosimilars are <more> widely available, how do you expect you might use them? | | | |

| **S5 Table: Patient acceptance of biosimilars** | | | | |
| --- | --- | --- | --- | --- |
|  | | Biosimilar and Biologic Naïve | Biologic originator patient in need of switch | Biologic originator patient not in need of switch for medical reasons |
| **% patients accepted without reluctance** | **Base** | **24** | **24** | **24** |
|  | Mean | 60.6 | 50.4 | 43.3 |
|  | Min | 5 | 5 | 5 |
|  | Max | 100 | 100 | 100 |
|  | StdDev | 27.78 | 27.86 | 27.45 |
| **% patients reluctant but accepted as no other choice** | **Base** | **24** | **24** | **24** |
|  | Mean | 21.0 | 25.6 | 29.4 |
|  | Min | 0 | 0 | 0 |
|  | Max | 60 | 60 | 65 |
|  | StdDev | 17.13 | 18.37 | 18.26 |
| **% patients refused but accepted bio‑originator** | **Base** | **24** | **24** | **24** |
|  | Mean | 10.2 | 15.8 | 18.0 |
|  | Min | 0 | 0 | 0 |
|  | Max | 40 | 70 | 50 |
|  | StdDev | 11.28 | 17.86 | 14.42 |
| **% patients refused biosimilar and bio-originator** | **Base** | **24** | **24** | **24** |
|  | Mean | 8.1 | 8.1 | 9.3 |
|  | Min | 0 | 0 | 0 |
|  | Max | 45 | 60 | 40 |
|  | StdDev | 9.20 | 12.92 | 10.62 |
| Question: Thinking of all the patients who had never before received a biologic originator or biosimilar (i.e. biologic/biosimilar naïve) for whom you tried to prescribe a biosimilar, what proportion. . . ?  Question: Thinking of all the patients who were receiving an originator biologic who, when they needed a switch of therapy you tried to prescribe a biosimilar, what proportion . . . ?  Question: Thinking of all the patients who were receiving an originator biologic who did not clinically need a switch of therapy, but you tried to prescribe a biosimilar, what proportion . . . ? | | | | |

| **S6 Table: Patient satisfaction (a) Satisfaction you are receiving the best treatment (b) Satisfaction with control of condition/symptoms by current treatment** | | | | |
| --- | --- | --- | --- | --- |
| 1. Are patients satisfied they are receiving the best treatment? | | | | |
|  | Base | | Biosimilar Patients | Bio-originator Patients |
| Base | 136 | | 70 | 66 |
| Yes, % (n) | 85 (115) | | 79 (55) | 91 (60) |
| No, % (n) | 7 (10) | | 13 (9) | 2 (1) |
| Ns, % (n) | 8 (11) | | 9 (6) | 8 (5) |
| 1. Patient satisfaction with current treatment of condition | | | | |
| Dissatisfied, % (n) | 1 (2) | 1 (1) | | 2 (1) |
| Neither satisfied nor dissatisfied, % (n) | 18 (24) | 27 (19) | | 8 (5) |
| Satisfied, % (n) | 58 (79) | 56 (39) | | 61 (40) |
| Very satisfied, % (n) | 19 (26) | 13 (9) | | 26 (17) |
| Ns, % (n) | 4 (5) | 3 (2) | | 5 (3) |
| Question: Considering all aspects of your current treatment (effectiveness, safety and quality) are you satisfied you are receiving the best treatment?  Question: Which option best describes your satisfaction with how well your current treatment is controlling your condition/symptoms? | | | | |

| **S7 Table: Patient concerns when first prescribed their treatment** | | | | | |
| --- | --- | --- | --- | --- | --- |
|  | Total | BioSN | BioSE | BioOA | BioOB |
| Base | 136 | 37 | 33 | 34 | 32 |
| Don't know enough about the drug, % (n) | 18 (25) | 24 (9) | 15 (5) | 12 (4) | 19 (6) |
| I think there are better medications, % (n) | 1 (2) | 3 (1) | - | 3 (1) | - |
| The medicine is too expensive, % (n) | 9 (12) | 11 (4) | - | 15 (5) | 9 (3) |
| Potential side effects, % (n) | 35 (47) | 41 (15) | 30 (10) | 32 (11) | 34 (11) |
| Potential long term problems, % (n) | 38 (51) | 41 (15) | 33 (11) | 38 (13) | 38 (12) |
| Doesn't help my symptoms overall, % (n) | 4 (6) | - | 3 (1) | 12 (4) | 3 (1) |
| I don't feel confident that this drug is tried and tested, % (n) | 4 (5) | 8 (3) | 3 (1) | - | 3 (1) |
| I think this version is a cheaper and less-effective version, % (n) | 2 (3) | 8 (3) | - | - | - |
| Other concern, % (n)s | - | - | - | - | - |
| No concerns, % (n) | 40 (54) | 35 (13) | 45 (15) | 41 (14) | 38 (12) |
| Ns, % (n) | 2 (3) | 3 (1) | 3 (1) | - | 3 (1) |
| Question: What concerns, if any, did/do you have about taking this medication at the times indicated at the top of the columns? | | | | | |
